# Inequities and psychiatry disability in transition among the elderly population from 1987 to 2006 in China: Data based on national surveys

**DOI:** 10.1097/MD.0000000000004779

**Published:** 2016-09-09

**Authors:** Zhenjie Wang, Ning Li, Chao Guo, Lei Zhang, Gong Chen, Xiaoying Zheng

**Affiliations:** a Institute of Population Research/WHO Collaborating Center on Reproductive Health and Population Science; b Laboratory of Neuroscience and Mental Health, Peking University, Haidian District, Beijing, People's Republic of China.

**Keywords:** China, older adults, psychiatry disability

## Abstract

The world will be facing huge population aged 65 and older, accounting for 13% of the total population in the future. Significant disabilities rates reflect an accumulation of health risks. Psychiatry disability is one of the most significant disabilities, because it manifests in cognitive, affective, and behavior disorders that limit one's daily life and restrict their participations. Very few studies have explored the 20 years associations between demographic factors and psychiatry disability among older people in China.

In this study, we investigated psychiatry disability transitional association behind China rapid development from 1987 to 2006 among the 60 and older population. Data used 2 nationally represented, population-based data from the China National Sample Surveys on Disability, conducted in 1987 and 2006. The sample size of the current study was 140,008 in 1987 and 354,859 in 2006, respectively. Associations between socioeconomic factors and psychiatry disability were determined by using a logistic regression model.

The prevalence of psychiatric disabilities increased from 1987 to 2006. In both surveys, the most common psychiatric disabilities were schizophrenia, schizotypal, and delusional disorders, and they presented the same associations with age increase. Socioeconomic inequities, such as current employment status and marital status, were associated with psychiatry disability in both surveys. These associations remained even after these 2 surveys were combined.

The rapidly rising prevalence of psychiatric disorders expected warrants strategies to reduce the burden of these disabilities among females and rural residents.

## 1 Introduction

The global population of people 65 years and older is expected to surpass 1 billion during the next 30 years, which will be 13% of the total population.^[1]^ In developed countries, the number of people aged 60 years and older is predicted to rise from 22% to 30% during the 1st 25 years of this century.^[2,3]^ In China, the prevalence of people aged 60 years and older accounted for 8% of the total population in 1990.^[4]^ That proportion increased to 12% in 2007.^[5]^ The number of population aged over 60 years and older is expected to increase to nearly one third of the total population of China in next 10 years, and over will exceed 440 million,^[4]^ making China one of the most aged societies in the world. Aging has a significant influence on disability trends.^[6]^ Higher disability rates among older people reflect an accumulation of health risks across a lifespan of disease, injury, and chronic illness.^[7]^ The disability prevalence in low-income countries is higher than in high-income countries, and higher among women than men.^[6]^ Psychiatric disabilities are some of the most significant disabilities, because they affect cognition and behavior. In 2006, approximately 5.8 million Chinese lived with psychiatric disabilities, and the national prevalence of psychiatry disability has tripled from 0.2% to 0.6% in 20 years.^[8]^ Another epidemiological survey on the prevalence of mental disorders in 4 Chinese provinces suggested that the rate was 8.0% for anxiety disorders, 5.9% for substance abuse disorders, and 0.8% for psychotic disorders among Chinese people aged 55 and older, respectively.^[9]^

Few studies have explored whether the associations between socioeconomic factors and psychiatric disabilities among people aged 60 years and older were consistent in China from 1987 to 2006. In this study, we investigated the associations between socioeconomic factors and risks for psychiatric disabilities in people aged 60 years and older, comparing data from 2 years, 1987 and 2006. We used data from 2 nationally representative population-based surveys on disabilities.^[10,11]^

## 2 Methods

### 2.1 Data source

Data were obtained from the1987 and 2006 China National Sample Surveys on Disability. All provincial administrative areas in mainland China were represented, excluding Hong Kong, Macau, and Chinese Taipei. Both surveys used multistage, stratified, random-cluster sampling, with probability in proportion to population, to derive nationally representative samples. Within each province, sampling strata were defined based on subordinate administrative areas, local geographical characteristics, or local gross-domestic product, to allow for regional variability. Within each stratum, a 4-stage sampling strategy was followed, involving 4 natural administrative units, and sampling was conducted with probability in proportion to cluster size. Researchers used the population and address information from the Ministry of Civil Affairs and Public Security in Beijing. The survey protocol and questions were reviewed by leading national and international experts. The sampling scheme was reviewed by experts from the Division of Statistics of the United Nations.^[10,11]^ The final sample size was 1,579,316 in the 1987 survey and 2,526,145 in the 2006 survey.^[10,11]^ The sampling ratio of subjects with disabilities, in the context of the total Chinese population, was 1.50 per 1000 people in 1987, and 1.93 per 1000 people for the 2006 survey.^[10,11]^ In the 1987 survey, 97.4% of the subjects were interviewed in person, 83.5% were interviewed in person for the 2006 survey.^[10,11]^ The 2 surveys were comparable in study design and administration.^[8,12,13]^

The surveys were approved by the State Council, and conducted in all province-level administrative regions of mainland China by the leading group of the China National Sample Survey on Disability and the National Bureau of Statistics. The surveys were conducted in accordance with statistical law in China. All respondents consented to the surveys.

### 2.2 Interviewing procedures and data quality

Before the surveys were administered, pilot studies were conducted in different provinces. There were strict quality control measures at every step, including the drafting of the sampling frame, field sampling, filling out of the questionnaires, checking of the returned forms, data input, and checking of data quality.^[10,11]^ Medical examinations were performed by a designated physician and were followed diagnostic manuals to make the final diagnosis and assess the severity of the disability, if any, and confirm its primary causes.^[8]^

After the field investigations were concluded, the teams made home revisits to conduct surveys for postsurvey quality checks and calculate errors in the surveys overall. The results of the quality checks showed that the omission rate of the resident population was 1.06 per 1000 persons in 1987 and 1.31 per 1000 persons in 2006; the omission rate of the disabled population was 1.16 per 1000 persons in 1987 and 1.12 per 1000 persons in 2006.^[10,11]^

### 2.3 Identification of psychiatry disability

The definition of psychiatry disability used was “Psychiatry disability refers to mental disorders lasting more than 1 year, which are manifested as cognitive, affective, and behavior disorders that limit one's daily life and restrict their participation.”^[10,11]^ The 1987 survey was based on the International Classification of Impairment, Disability, and Handicap.^[14]^ The 2006 survey design was based on the International Classification of Functioning, Disability, and Health.^[15]^

During data collection on psychiatry disability, trained field interviewers used a structured questionnaire about psychiatric disabilities. The questions included:

- Are you or your family members forgetful?
- Do you have difficulties in concentrating?
- Cannot you control your moods?
- Do you have strange behavior that is out of the ordinary?
- Are you addicted to alcohol or drugs?

Subjects who answered yes to any of the questions were referred to designated psychiatrist for further psychiatry disability confirmation. A designated psychiatrist performed medical examinations and followed diagnostic manuals to make the final diagnosis of the disability, if any, and confirm its primary causes of psychiatric disabilities.

Types of psychiatry disability were defined in the 1987 survey as follows: organic mental disorders; other mental disorders; schizophrenia, schizotypal, and delusional disorders; mood disorders; and epilepsy. Types of psychiatric disabilities were defined in the 2006 survey as follows: organic mental disorders; other mental disorders; schizophrenia, schizotypal, and delusional disorders; mood disorders; neurotic stress-related and somatoform disorders; behavior syndromes; disorder of adult personality and behavior; and epilepsy. Psychiatrists used the World Health Organization Disability Social Disability Screening Schedule in 1987^[16]^ and the World Health Organization Disability Assessment Schedule Phase II^[17]^ in 2006, as a scoring tool to assess the severity of the mental disability. Severity of the disability was classified into 4 categories: mild, moderate, severe, and extremely severe. Classification, the screening method, diagnostic method, and relevant scales on disabilities were all pretested in pilot studies with good reliability and validity.^[12,13,18]^

### 2.4 Study variables defined

We defined the status of psychiatry disability as binary, that is, yes or no; age group as 60 to 64, 65 to 69, 70 to 74, 75to 79, and 80+; gender as male or female; residential area as urban or rural; ethnicity as Han or others; education level as never attended school, primary school, junior high school, and above; marital status as never married, divorced/widowed, and married; household size as 1 to 3, 4 to 6, and 7 to 9 (persons/household); living arrangement as living with others or living alone; and currently employment status as employed or unemployed.

### 2.5 Statistical analysis

Our study population consisted of respondents aged 60 years and older. The 1987 survey had 140,008 subjects, including 680 cases of psychiatry disability. The 2006 survey had 354,869 subjects, including 3848 psychiatry disability cases. We calculated the age-adjusted prevalence of psychiatry disability through direct standardization by using the 2000 China population census as the standard.^[19]^ The different proportions between the 1987 and 2006 survey were tested by Chi-square test. The Mantel–Haenszel Chi-square test was used for trend association of categorical variables. A multivariable logistic regression model was used to calculate the adjusted odds ratios and 95% confidence interval. Statistical significance was declared if 2-sided *P* was <0.05. Statistical analyses were performed using SAS v. 9.2 (SAS Institute, Inc., Cary, NC).

## 3 Results

### 3.1 Characteristics of the subjects

Selected characteristics of the population under study are summarized in Table 1. In study, the prevalence of psychiatry disability was increased 2-fold from 1987 to 2006. The proportion of people over 75 years and older was higher in 2006 than in 1987 (24% and 19%, respectively). In surveys, female subjects, rural residents, people living with others, and Han nationality constituted the majority. In addition, structures of household size and education markedly changed. We also conducted multiple analyses within categorical variables (*P* value was adjusted by using the Bonferroni method). There were significant differences between primary school, no school, and junior high school.

**Table 1:**
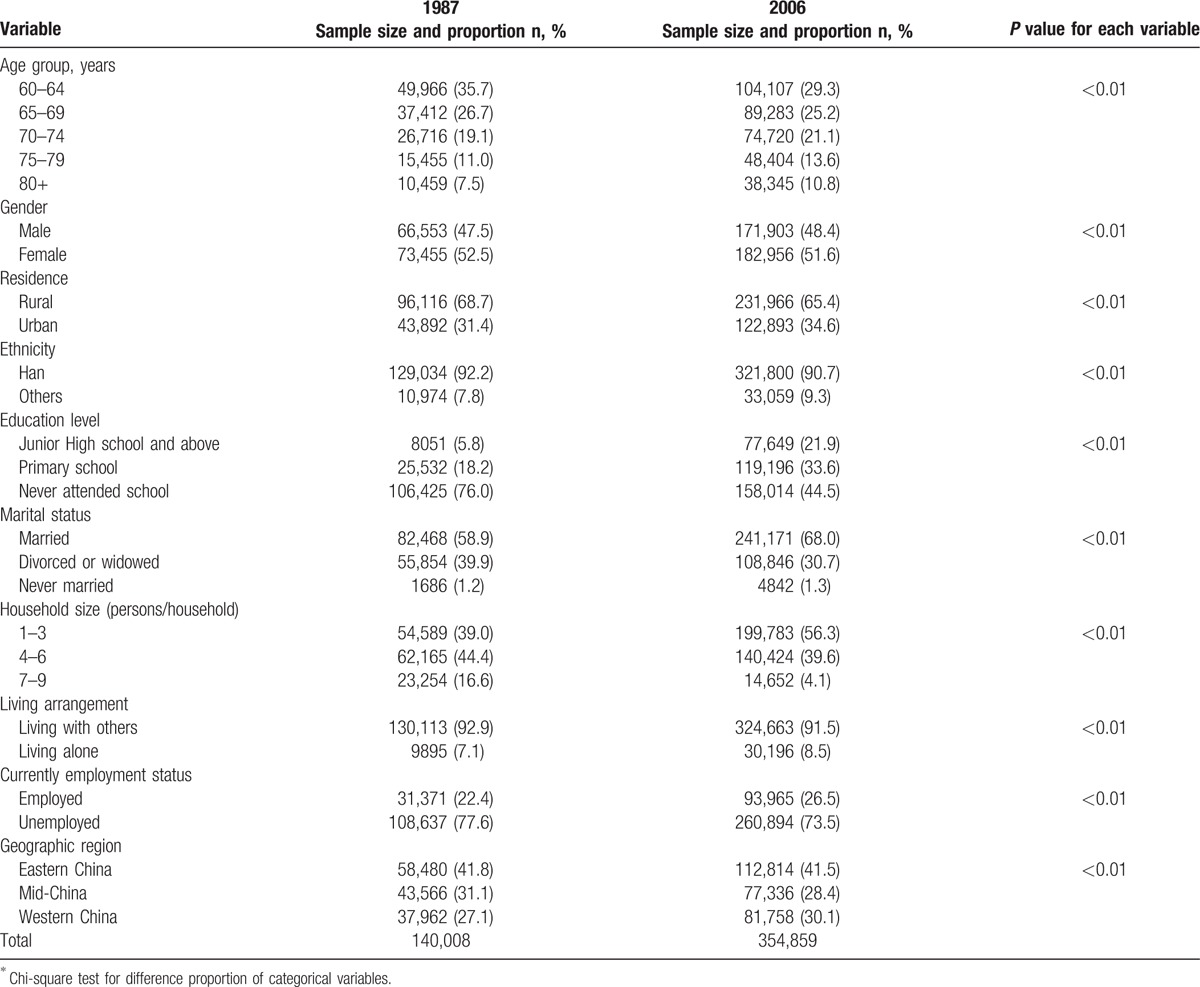
Demographic and socioeconomic characteristics in older population^∗^.

**Figure 1:**
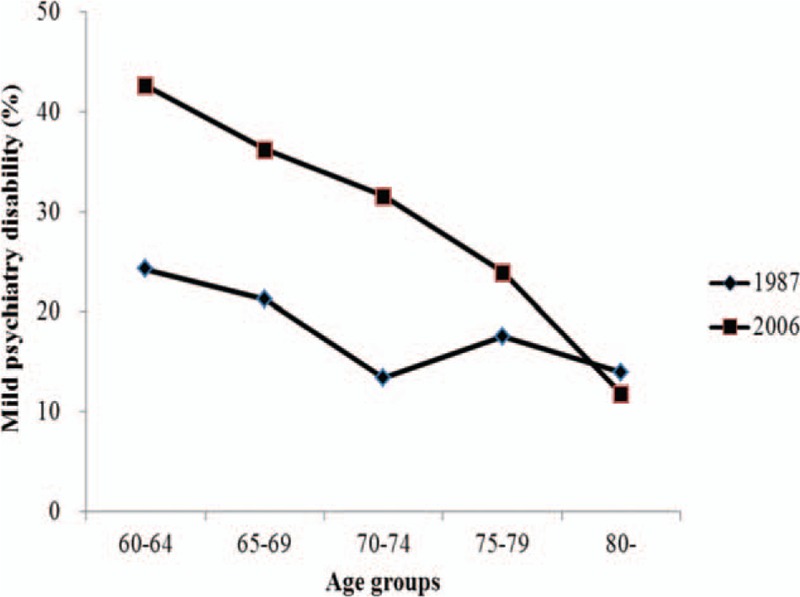
Proportion of mild psychiatry disability in 1987 and 2006 according to different age groups.

**Figure 2:**
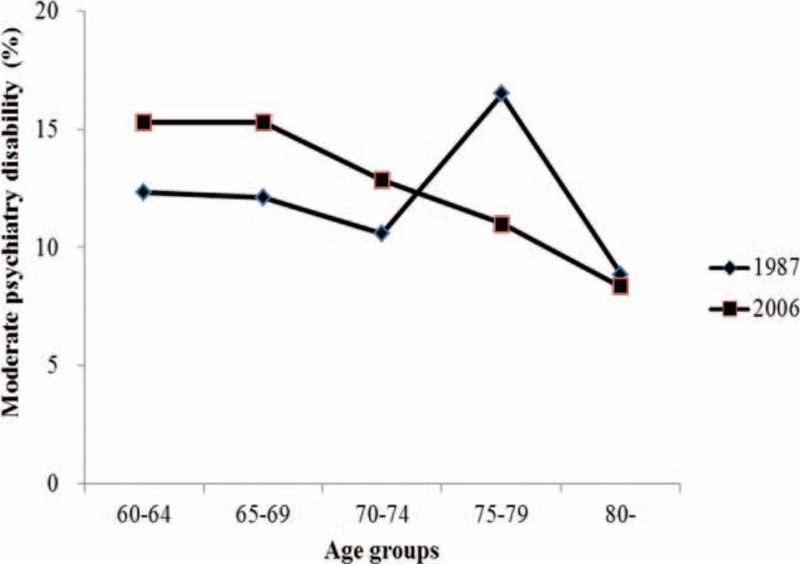
Proportion of moderate psychiatry disability in 1987 and 2006 according to different age groups.

**Figure 3:**
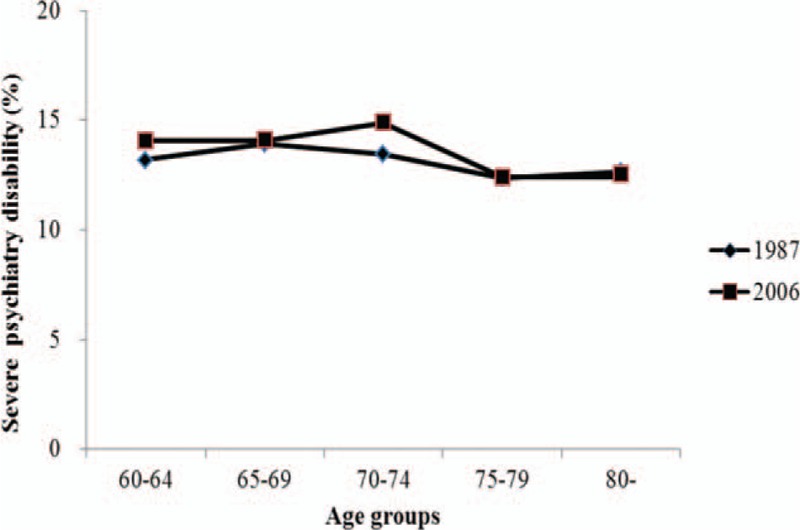
Proportion of severe psychiatry disability in 1987 and 2006 according to different age groups.

**Figure 4:**
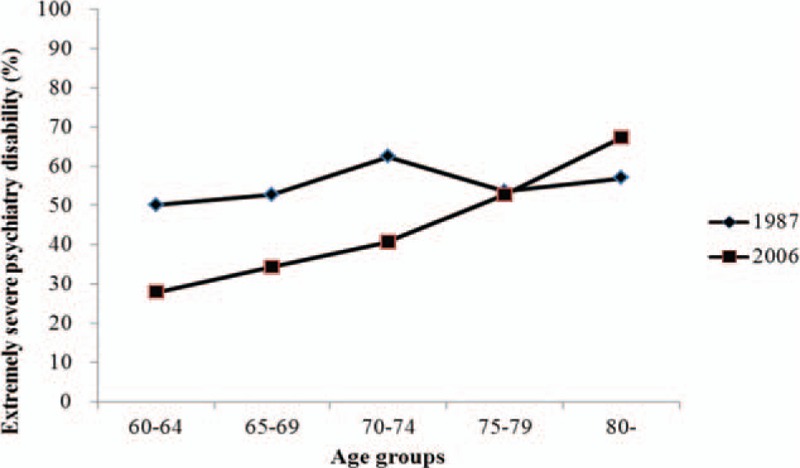
Proportion of extremely severe psychiatry disability in 1987 and 2006 according to different age groups.

In the 2006 survey, the proportions of mild and moderate psychiatry disability were negatively associated with age increase, but the proportion of extremely severe psychiatry disability was positively associated with age increase (Figs. 1–4). Table 2 presents psychiatry disability types in 1987 and 2006 by age groups. In both surveys, organic mental disorders and schizophrenia were positively associated with age increase, schizotypal, and delusional disorders were negatively associated with age increase, while other causes of psychiatry disability did not present a clear connection with age increase.

**Table 2:**
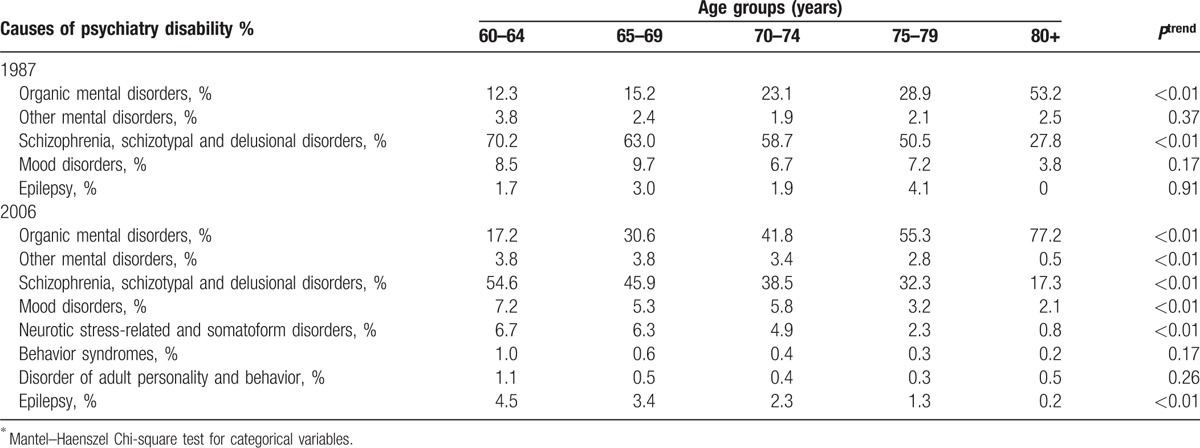
Distribution of psychiatry disability types by age groups^∗^.

### 3.2 Associations between socioeconomic factors and psychiatry disability

Logistic analyses showed material differences in association with demographical characteristics between the 1987 and 2006 surveys (Table 3). Age was the most important predictor of psychiatry disability. In the 2006 survey, which compared subjects with people aged 80 years and older, the probability of psychiatry disability decreased 21% in people aged 70 to 74 years, and 14% in those aged 65 to 69 years. In the 1987 survey, risk decreased by 40% in ages 70 to 74. After combining these 2 surveys, people aged between 65 to 69 and 70 to 74 years maintained the same association with psychiatry disability.

**Table 3:**
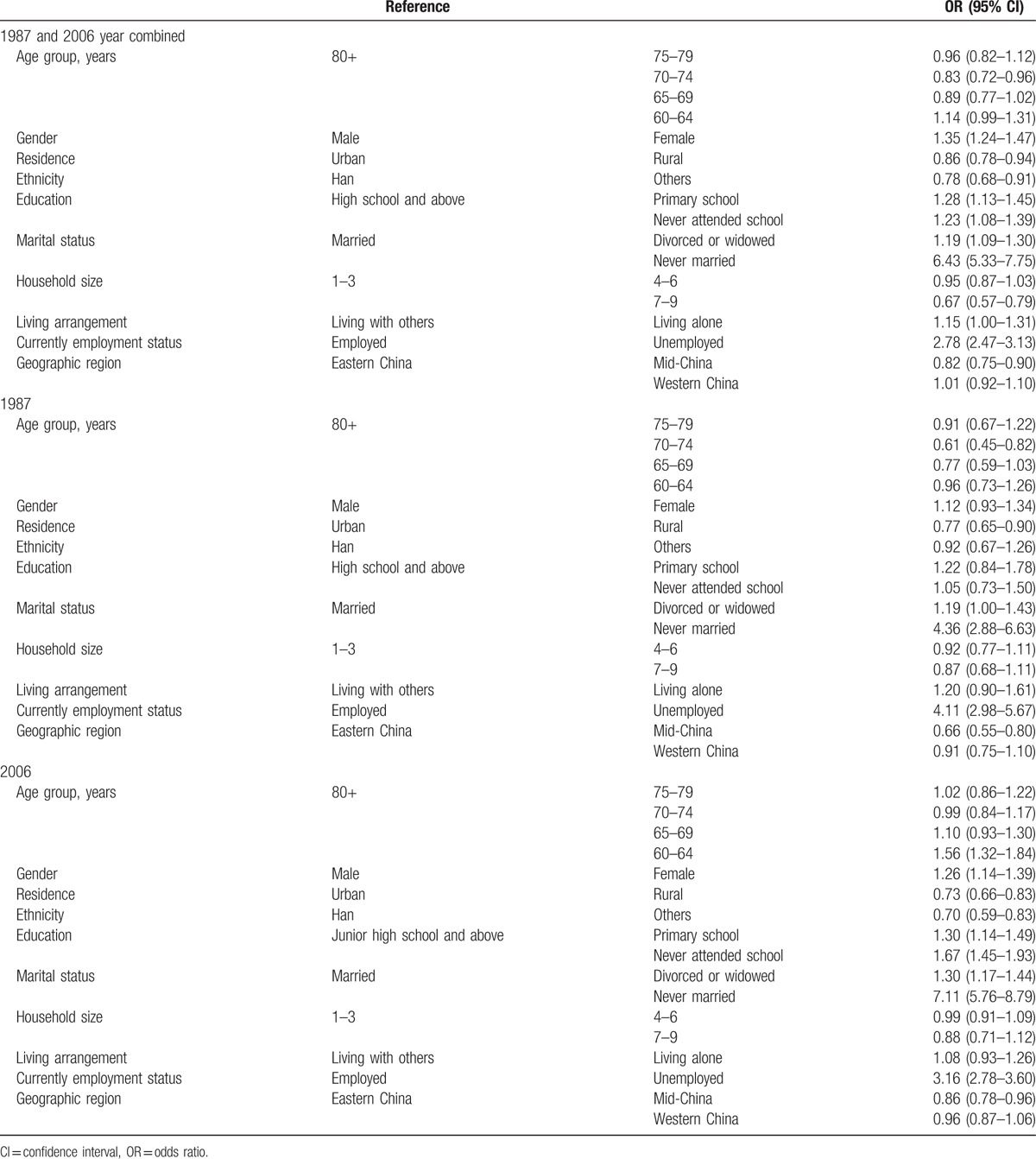
Associations of psychiatry disability in older people in China by demographical characteristics.

There was a significantly increased association between gender, education, marital status, and employment with psychiatric disabilities. Female was more likely to be at risk for psychiatric disabilities than male. In the 2006 survey, people with less education (i.e., those who had never attended school) were almost 1.6 times as likely to be disabled as those with higher education (high school and above). People who never married were over 4 times as likely to have psychiatry disability as those who married in both surveys. Psychiatric disabilities among those who were unemployed tripled in 2006 and quadrupled in 1987, compared to the employed. Similar associations were observed after these 2 surveys were combined.

We repeatedly analyzed the associations between socioeconomic factors and psychiatry disability by using stepwise logistic regression. In the 1987 survey, age groups (70–74, 65–59), gender, residence, education (primary school), marital status (divorced or widowed, never married), living arrangement, current employment status, and geographic region were contained in the final model. In the 2006 survey, age groups (65–69, 60–64), gender, residence, ethnicity, education (primary school, never attended school), marital status (divorced or widowed, never married), living arrangement, current employment status, and geographic region (mid-China) were contained in the final model. Age groups (70–74, 60–64), gender, residence, ethnicity, education (primary school, never attended school), marital status (divorced or widowed, never married), household size (7–9), living arrangement, current employment status, and geographic region (mid-China) were contained in the final model in the 1987 and 2006 combination model.

## 4 Discussion

### 4.1 Main findings and their significant findings

Using detailed personal interviews and professional examinations of psychiatry disability from the 1987 and 2006 nationally representative sample, we obtained valuable data on psychiatric disabilities among elderly Chinese people. Organic mental disorders, schizophrenia, schizotypal, and delusional disorders presented the same associations across age groups. We observed the same associations between socioeconomic inequities and psychiatric disabilities in both surveys, even when combined, as with previous studies. We also observed an association between gender and psychiatry disability, which was inconsistent with previous studies.

### 4.2 Comparisons with other studies and implications of findings

Among elderly people, the prevalence of psychiatry disability as diagnosed, based on performance, increased by nearly 2-fold in 20 years. There is no national Mental Health Act in China, and government has not given a mental health service system higher priority.^[20,21]^ China lacks qualified mental health professionals.^[20]^ There are 1.3 psychiatrists and 2.1 psychiatric nurses per 100,000 people.^[22]^ Slow development of specialized training and treatment of mental disorders as well as culturally rooted stigmas about mental disorders are also barriers to the improvement of mental health care in China.^[20,21]^ Moreover, aging has a significant influence on disability trends. The relationship is straightforward: there is a greater risk of disability at older ages, and national populations are aging at unprecedented rates. Compared with other studies, the disability prevalence was increasing among people 45 years and older in low- and high-income countries, especially among those aged over 55 years.^[6]^ In this study, not like in the WHO report, the prevalence of psychiatry disability was markedly increasing among people 70 years and older in both surveys, especially for the 2006 survey.

Elderly people may suffer from a number of mental and behavioral disorders, as the prevalence of some disorders increases with age.^[23]^ We observed that some types of psychiatry disability increase with age, such as organic mental disorders and undetected mental health disorders. Usually, the prevalence of epilepsy increases with age and the annual prevalence of epilepsy rose from 6.0 per 1000 people in those aged 60 to 64 years, to more than 7.7 per 100,000 people aged 85 and older. The prevalence of epilepsy across all age groups is 5.2 per 1000 people.^[24]^ In our study, however, the prevalence of epilepsy fluctuated from 8.0 per 1000 people for those aged 60 to 64 years to 2.6 per 1000 people aged 75 to 79 years in the 1987 survey. The similar prevalence trend of epilepsy also appeared in the 2006 survey. Neither of our survey results was consistent with previous studies.

Across the world, people with disabilities have poorer health outcomes, fewer educational achievements, less economic participation, and higher rates of poverty than people without disabilities.^[6]^ One explanation is that people with disabilities experience barriers in accessing services that many of us have long taken for granted, including health, education, employment, and transportation, as well as instant and never-ending information.^[6]^ Specifically, education inequalities are associated with disabilities. A study on education and disability conducted in Europe suggested that higher education serves to postpone or avoid disability among older people.^[25]^ Other studies have suggested that a gradient in disability was influenced by education, occupation, and material living standards.^[26,27]^ Results from these studies were consistent with findings from our current study. We also observed that people who lived in rural areas were more easily affected by psychiatric disabilities, compared to those living in urban areas.^[6]^ In our study, we observed a gender difference in elderly people, females were more vulnerable to psychiatry disability compared with males. The greatest gender gap usually occurs in midlife. There were no differences reported in childhood and few in the elderly.^[23]^ This interesting result may be attributed to genetic, biological, psychological, and/or social factors.^[23]^ Our findings confirm a similar pattern with previous suicide research in China.^[28]^ Phillips et al^[28]^ observed greater prevalence of psychiatric disabilities and suicide among adult, rural females in China. This finding may bring attention to a possible connection between psychiatry disability and suicide prevention among females in China.^[28]^

### 4.3 Strengths and limitations

This study provides a broad understanding of psychiatry disability and its relationship with key components of socioeconomic status from 1987 to 2006. A large, representative, population-based sampling covered all the provincial areas. In addition, every subject of the selected households was interviewed by interviewers face to face. A screen scale of disabilities was conducted by interviewers, and those suspected to be disabled were then examined and diagnosed by doctors. The present study has some weaknesses, such as the 1987 survey used the International Classification of Impairments, Disabilities, and Handicaps,^[14]^ and the 2006 survey used the International Classification of Functioning, Disability, and Health^[15]^ to classify disability. Both surveys used the Chinese word “Canji,” which means both handicap and disability, and helps to keep the consistency of the definition used in both surveys. Furthermore, although there were some differences in screening methods, diagnostic methods, and the classification of psychiatric between 1987 and 2006, they were comparable and presented good reliability and validity,^[12,13,18]^ it should be cautious for future studies. The 2 surveys’ questionnaires did not include all related confounding variables, such as history of chronic diseases and household income, which should be considered by future studies. Additionally, standardized quality control schemes were in place during the field implementation, such as training of the interviewers and cross-checking the returned survey responses by contacting survey participants to lessen response bias.^[29]^

## 5 Conclusion

China is experiencing social and economic transition, with psychiatric disabilities becoming a significant population health issue. Our results are of benefit for understanding psychiatric disabilities and associations between psychiatric disabilities and socioeconomic factors among elderly people in China. The rapidly rising prevalence of psychiatry disability and significant associations with socioeconomic factors suggested expected warrants strategies to reduce psychiatry disability, especial for females, and rural residents.

## Acknowledgments

The authors thank State Key Development Program of Basic Research of China (973No. 2007CB511901), Yang Zi Program of MOE, State Key Funds of Social Science Project (Research on Disability Prevention Measurement in China, No. 09&ZD072,) the Research Special Fund for Public Welfare Industry of Health (No. 201302008) as well as China Postdoctoral Science Foundation (Grant Number 2015M570004), and the Scientific Research Foundation for the Returned Overseas Chinese Scholars, State Education Ministry for the support.
